# Supplementary material for: Combining heterogeneous data sources for neuroimaging based diagnosis: re-weighting and selecting what is important
Source: Neuroimage. 2019 Jul 15;195:215–31. doi: 10.1016/j.neuroimage.2019.01.053 (PMC6547052; doi:10.1016/j.neuroimage.2019.01.053)
Supplement: supp_V3 [file mmc1.pdf]

Combining heterogeneous data sources for neuroimaging based diagnosis: re-weighting and selecting what is important

Michele Donini[a,*], João M. Monteiro[b,c], Massimiliano Pontil[a,c], Tim Hahn[d], Andreas J. Fallgatter[e], John Shawe-Taylor[c], Janaina Mourão-Miranda[b,f], for the Alzheimer's Disease Neuroimaging Initiative[†]

[a]*Computational Statistics and Machine Learning (CSML), Istituto Italiano di Tecnologia, Genova, Italy*
[b]*Max Planck University College London Centre for Computational Psychiatry and Ageing Research, University College London, UK*
[c]*Department of Computer Science, University College London, United Kingdom*
[d]*Department of Psychiatry and Psychotherapy, University of Münster, Germany*
[e]*Department of Psychiatry and Psychotherapy, University Hospital Tuebingen, Germany*
[f]*Centre for Medical Image Computing, Department of Computer Science, University College London, UK.*

Keywords: Multiple kernel learning, feature selection, neuroimaging.

Supplementary Material

In this section we present the results of the same experiments described in the main paper with the difference that the algorithms can use all the clinical information without any restriction. In the following the results for both the datasets, i.e. ADNI and Depression.

0.1. ADNI

The accuracy results for the ADNI dataset are presented in Table 1.

Figure 1 shows the assigned weights of the clinical information by using all the clinical features.

*Corresponding author
Email address: `michele.donini@iit.it` (Michele Donini)

† Data used in preparation of this article were obtained from the Alzheimer's Disease Neuroimaging Initiative (ADNI) database (`adni.loni.usc.edu`). As such, the investigators within the ADNI contributed to the design and implementation of ADNI and/or provided data but did not participate in analysis or writing of this report. A complete listing of ADNI investigators can be found at: `adni.loni.usc.edu/wp-content/uploads/how_to_apply/ADNI_Acknowledgement_List.pdf`

	Algorithm	Kernels	R	Bal. Acc. %
Baseline	Linear SVM	$\mathbf{C}$	1	68.73 ± 9.68
	SVM	$\mathbf{I} + \mathbf{C}$	1	84.80 ± 6.87
FS	SVM RFE	$\mathcal{V}$ & $\mathcal{C}$	$-$	86.93 ± 4.76
	SVM t-test	$\mathcal{V}$ & $\mathcal{C}$	$-$	86.47 ± 6.92
MKL	SimpleMKL	$\mathbf{I}$ & $\mathcal{C}$	36	84.44 ± 6.68
	EasyMKL	$\mathbf{I}$ & $\mathcal{C}$	36	84.78 ± 6.76
FW	SimpleMKL	$\mathcal{V}$ & $\mathcal{C}$	168165	Out of memory
	EasyMKL	$\mathcal{V}$ & $\mathcal{C}$	168165	88.80 ± 7.02
FWS	EasyMKLFS	$\mathcal{V}$ & $\mathcal{C}$	168165	96.14 ± 3.55

Table 1: ADNI Dataset: comparisons of 5 repetitions of a nested 10-fold cross-validation balanced accuracy using all the clinical information. The results are divided in 5 families: Baseline, Feature Selection (FS), standard Multiple Kernel Learning (MKL), Feature Weighting by using MKL (FW) and our method in Feature Weighting and Selection (FWS). R corresponds to the number of kernels used.

Finally, in Figure 2 it is possible to note the importance of the clinical data compared to the weight assigned to the voxel of the MRI images.

0.2. Depression

The accuracy results for the Depression dataset are presented in Table 2.

Figure 3 and 4 depict the assigned weights of the clinical information by using all the clinical features and the ration between the weight assigned to the clinical data with respect to the weight assigned to the different fMRIs.

	Algorithm	Kernels	R	Bal. Acc. %
Baseline	Linear SVM	$\mathbf{C}$	1	83.33 ± 15.71
	SVM	$\mathbf{I} + \mathbf{C}$	1	67.00 ± 14.87
FS	SVM RFE	$\mathcal{V}$ & $\mathcal{C}$	–	65.24 ± 11.34
	SVM t-test	$\mathcal{V}$ & $\mathcal{C}$	–	63.89 ± 10.32
MKL	SimpleMKL	$\mathbf{I}$ & $\mathcal{C}$	49	84.65 ± 12.88
	EasyMKL	$\mathbf{I}$ & $\mathcal{C}$	49	84.56 ± 13.02
FW	SimpleMKL	$\mathcal{V}$ & $\mathcal{C}$	713864	Out of memory
	EasyMKL	$\mathcal{V}$ & $\mathcal{C}$	713864	84.55 ± 12.19
FWS	EasyMKLFS	$\mathcal{V}$ & $\mathcal{C}$	713864	84.21 ± 10.72

Table 2: Depression Dataset: comparisons of 5 repetitions of a nested 10-fold cross-validation balanced accuracy using all the clinical information. The results are divided in 5 families: Baseline, Feature Selection (FS), standard Multiple Kernel Learning (MKL), Feature Weighting by using MKL (FW) and our method in Feature Weighting and Selection (FWS). R corresponds to the number of kernels used.

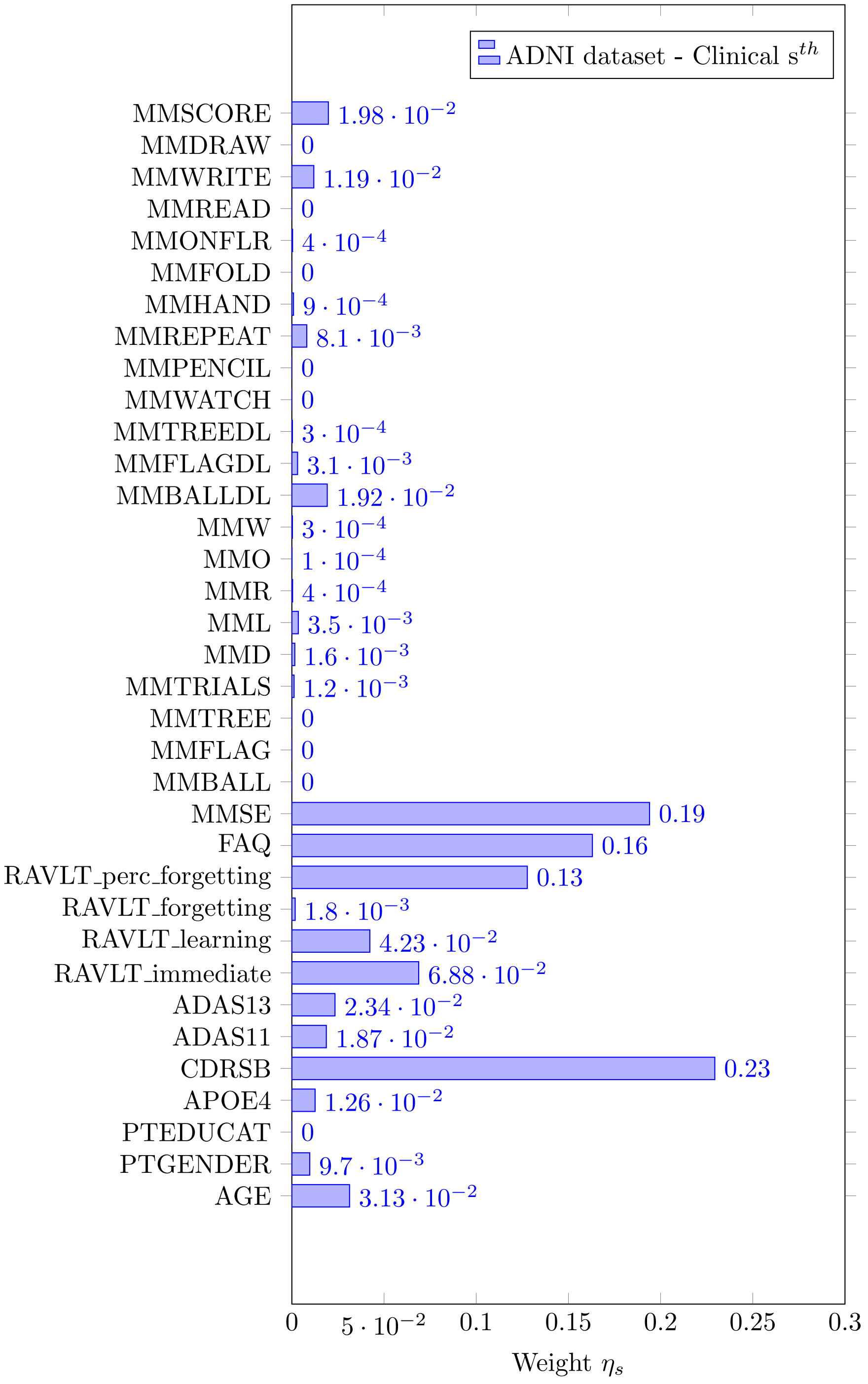

Figure 1: EasyMKL assigned weights for the all the clinical information for the ADNI dataset.

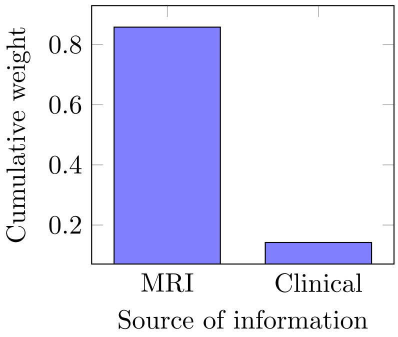

Figure 2: EasyMKLFS assigned weights for the different sources of information: MRI image and all the clinical measurements.

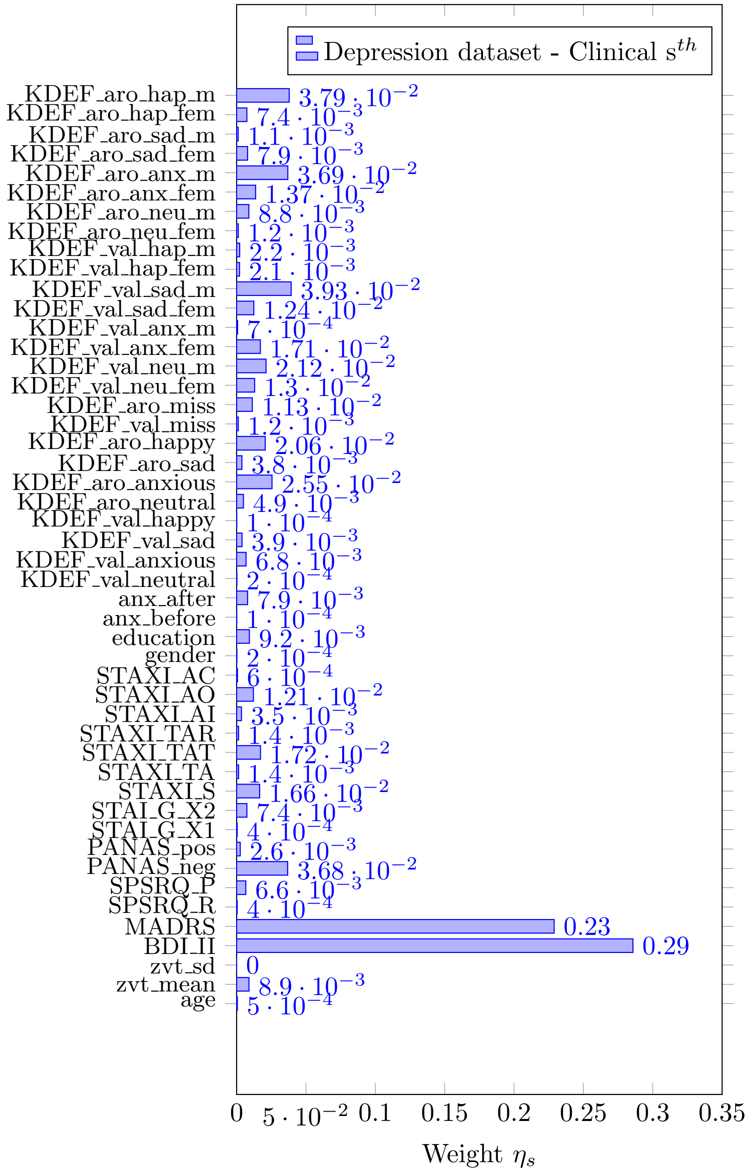

Figure 3: EasyMKL assigned weights for the clinical information for the Depression dataset.

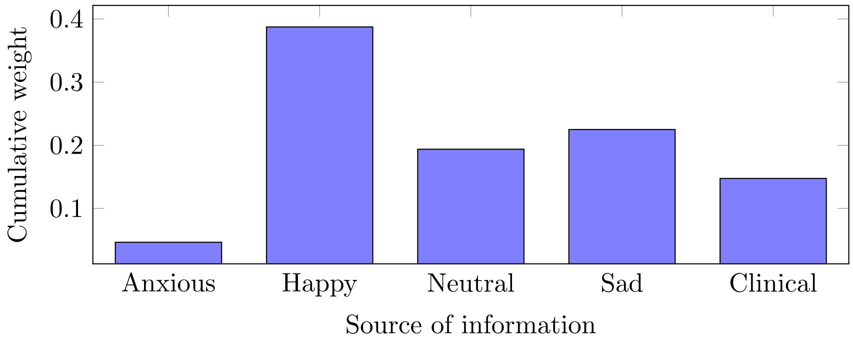

Figure 4: EasyMKLFS assigned weights for the different sources of information of the Depression dataset: Anxious image, Happy image, Neutral image, Sad image and clinical measurements.
